# A mapping of health education institutions and programs in the WHO African Region

**DOI:** 10.12688/aasopenres.13320.1

**Published:** 2021-12-14

**Authors:** Aaron N. Yarmoshuk, Pierre Abomo, Niamh Fitzgerald, Donald C. Cole, Arnaud Fontanet, Henry A. Adeola, Christina Zarowsky, Justin Pulford

**Affiliations:** 1 School of Public Health, University of the Western Cape, Bellville, South Africa; 2 Dalla Lana School of Public Health, University of Toronto, Toronto, Canada; 3 Centre for Capacity Research, Liverpool School of Tropical Medicine, Liverpool, UK; 4 Faculty of Arts and Science, University of Toronto, Toronto, Canada; 5 Centre for Global Health Research and Education, Institut Pasteur, Paris, France; 6 Conservatoire National des Arts et Métiers, Paris, France; 7 Division of Dermatology, Faculty of Health Sciences, University of Cape Town, Cape Town, South Africa; 8 Faculty of Dentistry, University of the Western Cape, Parow, South Africa; 9 CReSP-UMontréal, Université de Montréal, Montréal, Canada; 10 CIUSS Centre-Sud de Montréal, Université de Montréal, Montréal, Canada

**Keywords:** health education, Africa, medicine, public health, nursing, post-graduate, research, university, health professional programs

## Abstract

**Background:** Information on health education institutions is required for planning, implementing and monitoring human resources for health strategies. Details on the number, type and distribution of medical and health science programs offered by African higher education institutions remains scattered.

**Methods:** We merged and updated datasets of health professional and post-graduate programs to develop a mapping of health education institutions covering the World Health Organization African Region as of 2021.

**Results:** Nine hundred and nine (909) institutions were identified in the 47 countries. Together they offered 1,157 health professional programs (235 medicine, 718 nursing, 77 public health and 146 pharmacy) and 1,674 post-graduate programs (42 certificates, 1,152 Master’s and 480 PhDs). Regionally, East Africa had the most countries with multiple academic health science centres - institutions offering medical degrees and at least one other health professional program. Among countries, South Africa had the most institutions and post-graduate programs with 182 and 596, respectfully. A further five countries had between 53-105 institutions, 12 countries had between 10 and 37 institutions, and 28 countries had between one and eight institutions. One country had no institution. Countries with the largest populations and gross domestic products had significantly more health education institutions and produced more scientific research (ANOVA testing).

**Discussion:** We envision an online database being made available in a visually attractive, user-friendly, open access format that nationally, registered institutions can add to and update. This would serve the needs of trainees, administrators, planners and researchers alike and support the World Health Organization’s *Global strategy on human resources for health: workforce 2030*.

## Abbreviations

AHSCs              academic health science centres

AUF                  Agence Universitaire de la Francophonie

COHRED          Council on Health Research for Development

ECFMG            Educational Commission for Foreign Medical Graduates

GDPs                gross domestic products

HEIs                 health education institutions

HRH                 human resources for health

WDOMS          World Directory of Medical Schools

PGPs                post graduate programs

SANC              South African Nursing Council

SSA                 sub-Saharan Africa

UK                  United Kingdom

USA                United States of America

## Introduction

The shortage of health personnel in the World Health Organization African Region (WHO AFR ^1^) is well documented ^1– 4^. Equally well documented is the relatively low research output of the African continent relative to other regions ^5, 6^. IJsselmuiden *et al*. ^7^ mapped advanced public health programs in Africa and Mullan *et al.* ^8^ mapped sub-Saharan African medical programs over a decade ago. Klopper and Uys ^9^ produced a book on nursing education in Africa, but it included mainly Anglophone countries and is not available widely in African libraries ^2^. These three sources took important steps towards mapping health education institutions (HEIs) ^3^ but most have lagged behind advances. For example, Ethiopia increased the number of medical schools from five to 23 between 2003 and 2009 ^10^, yet the Mullan *et al.* ^8^ mapping listed only 12.

Objective Four of the WHO’s *Global strategy on human resources for health* ^11^ addresses the need to strengthen human resources for health (HRH) ^4^ data to improve “monitoring and accountability of national and regional strategies ….” The first milestone for this objective is, “[B]y 2020, all countries will have made progress to establish registries to track health workforce stock, education, distribution, flows, demand, capacity and remuneration” (Ibid. p.33]. An accessible, up-to-date mapping of health education throughout WHO AFR would help to reach one part of this milestone. It would assist institutions offering and considering offering programs, African students and planners in ministries of health and education, as well as granting agencies, institutions, and individuals interested in supporting HRH development throughout the region.

This paper presents a first joint mapping of institutions offering health education programs in WHO AFR and discusses issues concerning their distribution. It concludes by proposing a format that would allow the data set to be updated on an ongoing basis and accessed freely by all stakeholders.

## The WHO African Region

The authors chose to map WHO AFR for a number of reasons. First, this paper builds on two data sets that both stated they mapped “sub-Saharan Africa (SSA)” though included countries differed. Two, SSA is not a formal region of the world ^5^. Three, WHO AFR is the main UN agency for health in Africa. Of course, mapping health programs in all members of the African Union would have been preferable but the resources of the team were limited. . 

WHO AFR consists of 47 members of the African Union ^6^. The top 10 (21%) most populous countries account for 66% of the region’s population, led by Nigeria with 18.4% and Ethiopia with 10.3%. WHO AFR works in three official languages, listing 22 countries as English-speaking, 21 as French-speaking and four as Portuguese-speaking. The AU has five sub-regions: North, Southern, East, West and Central. ^7^. 

The economies of the countries range in size from approximately US$400 billion for Nigeria to US$400 million for São Tomé and Príncipe. Average per capita income ranges from US$16,434 (30,557 Int’l$ ^8^) in Seychelles to US$272 (744 Int’l$) for Burundi. South Africa is the most economically unequal member with a GINI Index of 63, Algeria the most equal with a GINI Index of 27.6. The Anglophone countries represent 67.5% of the GDP of WHO AFR, Francophone 25.7% and Lusophone 6.7%.

Current average health expenditure per capita ranges from 1,207 Int’l$ in Mauritius to 30 Int’l$ in the Central African Republic. Regarding human resources for health, Liberia has the fewest nurses per 1,000 people with 0.10 and South Africa the most at 3.52, Malawi the fewest physicians per 1,000 people with 0.02 and Mauritius the most with 2.02 and Sierra Leona has the fewest specialist surgical workforce per 100,000 with 0.13 and Seychelles the most with 48.57 ^9 12^. Centre for Capacity Research ^12^ includes a complete listing of the 47 countries of the region with key geographic, demographic, economic, health, human resources for health and research indicators, extracted from World Bank ^10^ and UNESCO sources ^11, 12^. 

## Methods

A team from the University of the Western Cape in South Africa and the University of Toronto in Canada developed the first data set of health professional programs (HPPs) in 2011. The second data set, of health post graduate programs (PGPs), was developed by a team from the Liverpool School of Tropical Medicine in the United Kingdom (UK) and the Institute Pasteur in France in 2017.

### Health professional programs

The first team mapped three HPPs (medicine, nursing and public health) in all WHO AFR countries except Algeria. Specifically, first-degree medicine programs (e.g. MD, MBBS, doctorat de medicine and diplôme d'État de docteur en medicine), nursing programs (in which a diploma or bachelor’s degree was earned) and public health programs (in which an MPH, MHSc or M.Med in Community Medicine or equivalent were earned) were mapped. Different campuses of the same institution were counted as separate institutions.

**Figure 1.**
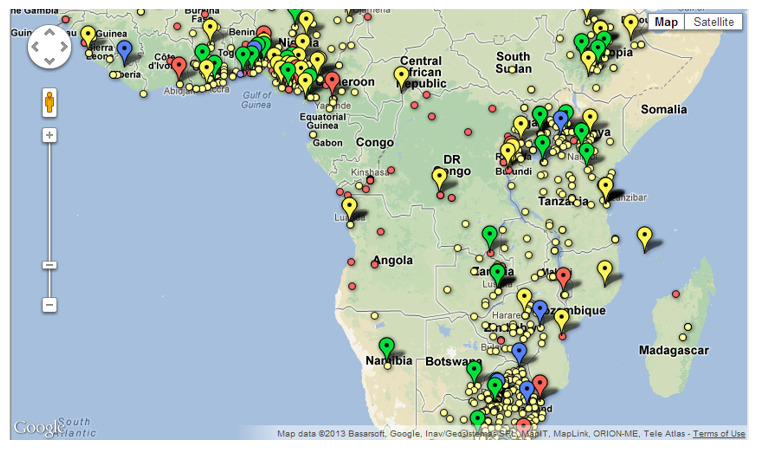
Screenshot of www.hppafrica.org.

The initial sources of data for the three types of HPPs were the Sub-Saharan African Medical Schools Study ^8^, the Health Training Institutions WHO AFRO Data 2005.xls ^13^ for nursing, and the Council on Health Research for Development (COHRED) database of African public health schools ^14^. The initial findings were complemented with information from *The Guide to Higher Education* ^13^, university web-sites and Wikipedia. In the latter two cases, searchers were conducted using the specific names of universities already identified and the terms “medical”, “nursing” or “public health” and “schools” or “program” (mes) and “Africa”. All data was entered into a MS Excel spreadsheet. The findings were analysed using SPPS and presented at two conferences ^15, 16^ and on a website ^17^ (see Figure 1).

### Post-graduate programs

The second team mapped four groupings of PGPs in SSA countries: 1) medical sciences; 2) biomedical sciences; 3) public health; and 4) ‘other’ health-related disciplines. “Medical sciences” consisted of second degrees, fellowships and diplomas in clinical fields including dentistry, medicine, nursing and pharmacy. “Biomedical sciences” consisted of life science programs directly relevant or applied to medicine. “Public health” included programs examining populations health and health promotion, for example epidemiology, biostatistics and nutrition. “Other” included all other health-related programs, for example health economics and hospital management.

Information was gathered online from March to August 2017 using: institutional registries; websites of HEIs in SSA; websites of the Ministries of Education and Health (to identify universities and schools of health training); websites of national accreditation bodies for health diplomas; and online registries such as the World Directory of Medical Schools ^18^ and information available from the Agence Universitaire de la Francophonie (AUF) ^19^ and the Foundation for Advancement of International Medical Education and Research (FAIMER) ^20^. Google searches used the names of countries or institutions and the keywords "Health Education in sub-Saharan Africa", "Health Education Institutions in sub-Saharan Africa", "Health Education in sub-Saharan Africa", and "Opportunities for Higher Education in sub-Saharan Africa". The findings were posted on the web-site of the Liverpool School of Tropical Medicine ^21^ and included in a written report ^22^.

### Updating and merging into a common data set

The HPP data set was updated and expanded, as practicable. The *World Directory of Medical Schools ^23^* (MDOMS) was accessed to update medical schools. Since only schools that have applied for certification from the Educational Commission for Foreign Medical Graduates (ECFMG) in the United States (US) were included in MDOMS, we included some unlisted medical programs, ^24^ in particular some non-anglophone programmes identified from other sources. Similarly, the HPP dataset had nursing programs not included in the South African Nursing Council (SANC) lists of accredited nursing education institutions available online ^25^ and some institutional names and/or locations (town or city) had changed. South African programs not recognized by the SANC were deleted and institutions names and cities were updated. Pharmacy programs were added using information from the International Pharmaceutical Federation ^26^ and national online sources, such as the Ghana Pharmacy Council web-site of accredited programs ^27^. In addition, Algerian institutions with medicine and pharmacy programs were added (not nursing nor public health programs, however) and PGPs were added for only one Algerian institution ^28^. The HPP and PGP data sets were merged manually to create a common data set with the number of institutions that had health education programs in medicine, nursing, public health, pharmacy and/or the number of PGPs each HEI offered in the health sciences - see Centre for Capacity Research ^14^. 

### Analyses

We produced two MSExcel tables: one of HEIs with binary “yes” or “no” columns for medicine, nursing, public health and pharmacy programs ^14^; and another listing all 47 WHO AFR countries and the total number of each HPPs, PGPs, HEIs, academic health science centres (AHSCs) and selected indicators from ^12^. AHSCs, institutions with a medical school and at least one other health professional program and a teaching hospital, were highlighted because they have the tripartite mission of providing education, conducting research and performing service, important for sustaining advancement in the health sciences ^15, 16^.

HPP, PGP and other country indicators were analyzed using SPSS27, including frequencies, crosstabs and analysis of variance (ANOVA). For the ANOVA analyses countries were grouped into strata of institutions per country (quartiles for HEIs and terciles for AHSCs) to compare means (SD) of population, GDP, GDP per capita (current), GINI Index, current health expenditure, life expectancy at birth, physicians - per 1,000 people (2010–17), and publications by field of science (total and medical sciences) across strata ^29 12^.

## Institutional and program findings

In total, 909 institutions together offered 1,176 health professional programs (235 medicine, 718 nursing, 77 public health and 146 pharmacy) as of July 2021 ^30^, and 1,641 post-graduate clinical and research programs (42 certificates, 1,149 Master’s and 480 PhDs) were offered at 183 of the 909 HEIs. South Africa, Nigeria, Kenya, Ethiopia and Ghana housed 482, or 52.7%, of the institutions (see Table 1). Of the PGPs, Medical Science programs were the most numerous with 851 (51.9%), followed by Biomedicine with 286 (17.4%), Public Health (including nutrition and environmental health) with 271 (16.5%), Nursing with 62 (3.8%), Dentistry with 47 (2.9) and Pharmacy with 45 (2.7%). São Tomé and Príncipe was the only country without any health education program perhaps as it is the only WHO AFR country with a GDP below US$1 billion, at US$422.3 million.

**Table 1.** Number of Health Education Institutions (HEIs), Post-Graduate Programs (PGPs) and Academic Health Science Centres (AHSCs) by sub-Region and Country, ranked within sub-region by country number of HEIs.

Sub-Region	Country	Number of HEIs	Number of PGPs	Number of AHSCs
**Central** 9 countries 149,487,474 pop (14.1% of region)	Cameroon	33	52	5
	Congo, Dem. Rep (DRC)	31	30	2
	Burundi	12	4	2
	Gabon	3	2	1
	Chad	3	5	0
	Congo, Rep	1	8	0
	Central African Republic	1	2	1
	Equatorial Guinea	1	0	0
	São Tomé and Príncipe	0	0	0
	**Central sub-totals**	**85**	**103**	**11**
**Eastern** 11 countries 314,607,642 pop (29.6% of region)	Kenya	76	178	7
	Ethiopia	65	136	27
	Tanzania, United Republic of	53	59	7
	Uganda	37	56	7
	Madagascar	10	32	1
	Rwanda	8	17	1
	Eritrea	6	2	0
	Mauritius	5	0	2
	South Sudan	5	0	1
	Comoros	2	0	0
	Seychelles	1	0	0
	**Eastern sub-totals**	**268**	**480**	**53**
**Northern** 2 countries 46,631,748 pop (4.4% of region)	Algeria	12	28	10
	Mauritania	4	2	1
	**Northern sub-totals**	**16**	**30**	**11**
**Southern** 10 countries 175,966,205 pop (16.5% of region)	South Africa	182	596	8
	Zimbabwe	34	25	1
	Zambia	28	15	6
	Malawi	15	2	1
	Angola	11	0	3
	Mozambique	7	1	3
	Botswana	6	1	2
	Namibia	4	5	1
	Lesotho	4	0	0
	Eswatini	3	1	0
	**Southern sub-totals**	**294**	**646**	**25**
**Western** 15 countries 376,793,379 pop. (35.4% of region)	Nigeria	105	202	32
	Ghana	64	45	5
	Niger	14	26	1
	Senegal	11	36	3
	Benin	5	24	2
	Liberia	7	7	1
	Cabo Verde	7	0	0
	Mali	5	3	1
	Gambia, The	6	5	2
	Côte d'Ivoire	5	30	1
	Burkina Faso	4	13	1
	Guinea	4	17	2
	Sierra Leone	4	1	2
	Togo	3	6	1
	Guinea-Bissau	2	0	0
	**Western sub-totals**	**246**	**415**	**54**
**Overall Totals**		**909**	**1674**	**154**

Anglophone countries had the most institutions overall and on a per capita basis [718 (79%)], although they account for 60.5% of WHO AFR’s population. Francophone countries had 164 institutions (18%) and Lusophone had 27 institutions (3%) but represent 33.6% and 5.9% of WHO AFR’s population, respectively. Anglophone country HEIs had more PGPs than did Francophone, 1,353 (81%) to 320 (18%), respectively. Ten countries had 114 of the 154 (74%) of the 154 AHSCs (see Table 1).

The ownership of 710 (78.1%) institutions was identifiable: 448 (63.1%) were publicly owned; 254, (35.9%,) were privately owned; and seven (1.0%) had joint public/private ownership. The date institutions were founded, or the year programs began, was found for 404 (44.4%) of cases. Of these, 152 (37.6%) were founded in 2000 or later. Just over half, 51.5%, of the private institutions were founded since 2000 compared to approximately one-third, 31.9%, of the public institutions.

**Figure 2.**
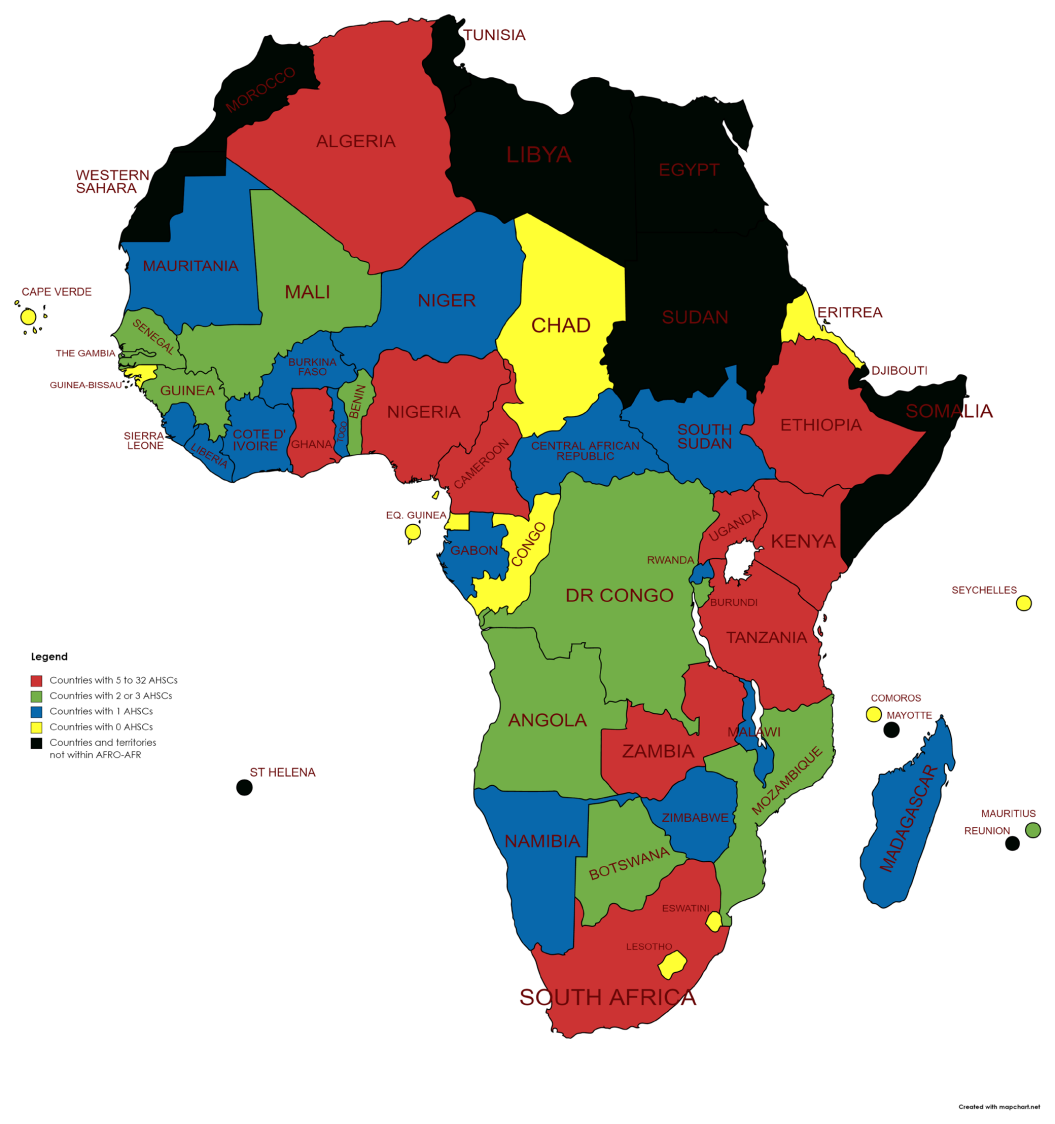
Map of WHO AFR Countries with range of Academic Health Science Centres (AHSCs).

Although the data are substantially skewed (SD > mean for many categories), there were statistically significant differences in indicators across a number of HEI strata (see Table 2). Countries with fewer institutions had a statistically significant lower population, GDP, total science publications and medical science publications (Tukey post-hoc test). A similar pattern was observed across terciles for AHSCs (0, 1, and >1 AHSC per country) ^31^ (see Figure 2).

**Table 2.** Country indicator values, by quartiles of numbers of Health Education Institutions per country.

Indicator	#HEI Quartile	Number of Countries	Indicator Mean (SD)	ANOVA F (p value)
**Population-Total in 2018** **(in millions)**	1 (0 to 3 HEIs)	11	3.7 (4.6)	11.3 (.000)
	2 (4 to 6 HEIs)	14	8.9 (7.8)	
	3 (7 to 15 HEIs)	11	19.5 (12.3)	
	4 (16 to 182 HEIs)	11	62.2 (52.7)	
	Overall	47	22.6 (34.3)	
**GDP (current US$) in 2018** **(in billions)**	1 (0 to 3 HEIs)	11	6.3 (5.8)	5.4 (.003)
	2 (4 to 6 HEIs)	14	12.2 (10.5)	
	3 (7 to 15 HEIs)	11	33.3 (55.1)	
	4 (16 to 182 HEIs)	11	112.0 (135.6)	
	Overall	47	39.1 (80.4)	
**Publications in All Fields of** **Science (2014)**	1 (0 to 3 HEIs)	11	42.7 (44.3)	3.1 (.035)
	2 (4 to 6 HEIs)	13	121.9 (95.2)	
	3 (7 to 15 HEIs)	11	332.5 (663.0)	
	4 (16 to 182 HEIs)	11	1544.5 (2,628.0)	
	Overall	46	493.5 (1,414.7)	
**Publications by Field of** **Science - Medical Sciences** **(2014)**	1 (0 to 3 HEIs)	11	9.4 (12.3)	5.2 (.004)
	2 (4 to 6 HEIs)	13	27.8 (21.2)	
	3 (7 to 15 HEIs)	11	38.4 (38.2)	
	4 (16 to 182 HEIs)	11	296.1 (405.3)	
	Overall	46	90.1 (225.2)	

## Discussion

Although the number of HEIs/country was associated with higher country populations, HEIs in WHO AFR were not equally distributed: over half of the institutions (54.1%) were located in five countries that together have only 41.8% of the WHO AFR population. These five countries (South Africa, Nigeria, Kenya, Ethiopia and Ghana) accounted for 54.5% of the region’s GDP (current US$) in 2018, consistent with our finding that GDP was also associated with number of HEIs and AHSCs. The uneven distribution of PGPs in WHO AFR was more marked, with only three countries (South Africa, Nigeria and Kenya) housing 58.3% of the PGPs. Such a concentration of medicine and health science PGPs is consistent with the findings of Adams, King ^17^ regarding scientific hubs in Africa. Algeria has a large number of AHSCs and likely has a large number of PGPs too, as the one university we collected data for had 28 PGPs. However, North Africa is not well represented in WHO AFR with only two members. Egypt, the research hub country in the sub-region ^17^, is a member of the WHO Eastern Mediterranean (WHO EMR) not WHO AFR.

The relatively low number of Francophone and Lusophone HEIs in the mapping is consistent with these groups of countries having lower GDPs per capita than Anglophone countries. As institutions in the non-Anglophone countries may have larger student bodies, information on the number of graduates per HEI per year would be useful. Bilingual Cameroon had the most AHSCs and PGPs of non-English countries (although it is officially bilingual in terms of UN languages). It is identified as having “significant relative productivity” in West Africa by Adams *et al*. ^18^. 

East Africa has the greatest diversity in terms of countries with multiple AHSCs (four countries with 7 or more AHSCs) and three countries with 53 or more PGPs, likely an important strength for the sub-region. Although Ethiopia, Kenya and Tanzania have the most HEIs and PGPs in the region, one of the best known AHSCs in the region, Makerere University, is in a fourth country (Uganda) and a relatively young and innovative institution, the University of Global Health Equity (UGHE), is in a fifth country (Rwanda). This subregion has long been the favourite of international university partners and donors reflected in its disproportionally high number of international partnerships ^19^.

Although such variation may demonstrate untenable inequities in human resources for health, concentration alone may not be problematic. Trainees from other African countries often attend and HEIs often support the development of HPPs and PGPs elsewhere. For example, the development of the post-graduate Ophthalmology program at the University of Nairobi in 1978 through a partnership with Ludwig Maximilian University of Munich started training non-Kenyans a few years after it was established. By 2013 167 students had graduated, 57 of them were from 16 other WHO AFR countries ^20^. The Nairobi program helped developed other PGPs in East Africa ^21, 22^. The importance of international partnerships for African institutions for research output and research capacity strengthening is often identified by researchers ^20, 23– 25^. At the same time, it has been argued that the growth in HEIs in WHO AFR in the first two decades of this century coincides with the growth in research on the continent, with increasing “autonomous research output” and research self reliance [ 18, p. 550]

As noted, our work suffers limitations: not including the nursing, public health or most PGPs of Algerian HEIs nor PGPs of Lusophone countries; lack of universal information about accreditation; and missing details on some institutions (e.g. date founded). Recently established institutions or programmes are also likely missing. Although our common data set is likely the most comprehensive of its kind currently, several challenges remain: a) to establish a managed, open-source, on-line, Wiki-like database that institutions can access to update their information and new institutions can add their details b) to develop a visually attractive, user-friendly web-site so trainees, researchers, administrators and other interested parties can access information they desire easily; and c) to ensure that all programs and institutions listed are registered in their country. We look forward to collaboration to develop this potentially useful resource. 

## Data availability

### Underlying data

Harvard Dataverse: Annex 1 - Master List of WHO-AFR Countries with Indicators, https://doi.org/10.7910/DVN/JHVEKJ ^12^. 

Harvard Dataverse: Annex 2 - Master List of HEIs, https://doi.org/10.7910/DVN/Q8CNY3 ^14^.

Data are available under the terms of the Creative Commons Zero "No rights reserved" data waiver (CC0 1.0 Public domain dedication).

## Footnotes

^1^ This paper refers to WHO AFR instead of WHO AFRO as the former refers to the WHO African Region as a whole whereas WHO AFRO refers to the World Health Organization Regional Office for Africa in Brazzaville, Congo.

^2^ Togo and Niger are the only francophone countries included. No Lusophone countries are included. On 27 Feb 2017 - https://www.worldcat.org/ - listed the book as being available at 501 libraries worldwide, although only 3 (0.6%) of them were in Africa - all in South Africa. On 11 June 2020, WorldCat listed the book as being available in 962 libraries worldwide, 14 (1.4%) in Africa - 11 in South Africa and 1 each in Botswana, Namibia and Nigeria.

^3^ HEI being institutions that create human resources for health (HRH), see footnote 4, below.

^4^ HRH includes “… all people primarily engaged in actions with the primary intent of enhancing health”, including health service providers and health management and support workers. This also includes health researchers. WHO, The world health report 2006: working together for health. 2006, Geneva, Switzerland: World Health Organization, p. xvi.

^5^ de Haldevang, M. Why do we still use the term “sub-Saharan Africa”? 2016 [accessed 1 September 2016]; Available from: http://qz.com/770350/why-do-we-still-say-subsaharan-africa/.

^6^ Seven other AU members are members of the WHO Regional Office for the Eastern Mediterranean (WHO-EMRO). The Sahrawi Arab Democratic Republic, or Western Sahara, is a full AU member but not a WHO member, as it is categorised as a Non-Self-Governing Territory by the United Nations.

^7^ We decided to use these five sub-regions instead of WHO AFRO’s three Inter-country Support Teams (ISTs) - https://www.afro.who.int/about-us/organizational-structure.- units for some analysis because they are more granular.

^8^ Int’l$ stands for current international dollars and presents GDP per capita reflecting purchasing power parity (PPP).

^9^ The most recent figure for each country was taken between the years 2010 and 2017.

^10^ World Bank. DataBank. 2020 [Accessed 14 May 2020]; Available from: https://databank.worldbank.org/.

^11^ UNESCO. UNESCO Institute for Statistics (UIS). 2020 [Accessed 15 May 2020]; Available from: http://data.uis.unesco.org/.

^12^ UNESCO, UNESCO science report: towards 2030. 2015, UNESCO: Paris, France.

^13^ This data set was received from the The Sub-Saharan African Medical Schools Study (SAMSS) at George Washington University. To our knowledge it is no longer publicly available. The dataset can be obtained by contacting the corresponding author.

^14^ COHRED. African Schools of Public Health (AfriHealth). [Accessed 1 September 2011]; Available from: www.cohred.org.

^16^ Yarmoshuk, A., *et al*. Mapping of Health Professional Programs in sub-Saharan Africa. 2012; Available from: https://www.researchgate.net/publication/341833320_Mapping_of_Health_Professional_Programs_in_sub-Saharan_Africa. Conference: Learning About Research Capacity StrengtheningAt: Cape Town, South Africa.

^17^ www.hppafrica.org. Note: the mapping feature on the web-site ceased to function in December 2019 when Google ceased offering Google Fusion.

^18^ WFME and FAIMER, World Directory of Medical Schools. World Federation for Medical Education and the Foundation for Advancement of International Medical Education and Research. 2014-2019. [Accessed in 2019, 2020 and 2021.] Available from: https://www.wdoms.org/.

^19^ L’Agence Universitaire de la Francophonie. L’Agence Universitaire de la Francophonie (AUF). 2017 [Accessed 2017; Available from: https://www.auf.org/.

^20^ Foundation for Advancement of International Medical Education and Research. Foundation for Advancement of International Medical Education and Research. 2017 [Accessed various dates in 2017]; Available from: https://www.faimer.org/.

^21^ http://www.lstmed.ac.uk/research/centres-and-units/capacity-research-unit-cru/our-projects/deltas-learning-research

^22^ Abomo, P., *et al*. DELTAS Africa Learning Research Programme: A list of postgraduate training programmes in Medical and Health Sciences provided by Higher Education Institutions in sub-Saharan Africa. 2017 [Accessed 11 July 2019]; Available from: https://www.lstmed.ac.uk/research/centres-and-units/capacity-research-unit-cru/our-projects/deltas-learning-research.

^23^ https://www.wdoms.org/ (accessed July 2021).

^24^ We are grateful to William Burdick for bringing this to our attention.

^25^ SANC. Universities Accredited To Offer The New Nursing Programmes (undated); SANC. Lists of Accredited Nursing Education Institutions (undated); SANC. Private Nursing Education Institutions Accredited To Offer The New Nursing Programmes With Effect From 2020: December 2019 Update. 2019; SANC. Private Nursing Education Institutions That Are Still Operating "Legacy" Programmes: March 2020 Update; SANC. Public Colleges Accredited To Offer The New Nursing Programmes With Effect from 2020: December 2019. 2019 15 May 2020 [cited 2020 14 May]; SANC. Public Nursing Education Institutions That Are Still Offering Legacy Qualifications 2018-02-01. 2018 2018-02-01 [Accessed 14 May 2020]; Available from: https://www.sanc.co.za/.

^26^ FIP. Official World List Of Pharmacy Schools. 2019. [Accessed 11 May 2020]; Available from: http://aim.fip.org/world-list-of-pharmacy-schools/?region=Africa&country=&show=AIM.

^27^ PCGhana. Accredited Pharmacy Programmes and Institutions. [Accessed 15 May 2020]; Available from: https://pcghana.org/.

^28^ It wasn’t practicable to be more comprehensive with programs in Algeria due to limited resources.

^29^ The data for Annex 1 were obtained from World Bank (2020), UNESCO (2015) and UNESCO (2020). See footnotes 10, 11 and 12.

^30^ As the data was collected over a number of years it is likely that some programs are missing while others may no longer be offered.

^31^ Details available on request from corresponding author.
