# Supplementary material for: A mapping of health education institutions and programs in the WHO African Region
Source: AAS Open Res. 2021 Dec 14;4:55. [Version 1] doi: 10.12688/aasopenres.13320.1 (PMC10891426; doi:10.12688/aasopenres.13320.1)
Supplement: Peer review history

# Reviewer report: Reviewer response for version 1

**DOI:** 10.21956/aasopenres.14450.r30485

**Published:** 2024-02-23

**Authors:** Angelique Wildschut

**Affiliations:** 1 Sociology, University of Pretoria, Pretoria, Gauteng, South Africa; 2 Human Sciences Research Council, Pretoria, South Africa

The paper aims to contribute towards establishing a comprehensive mapping of health education institutions and programs in the WHO African region. The team combined information on health professional programs constructed in 2011 and another data set of health post graduate programs developed in 2017. Information on health professional programs drew from 2005 data and were complemented with information from other sources (including Wikipedia). For post-graduate programmes the dataset was based on information gathered online from March to August 2017. In terms of validity and reliability, it would thus appear that the dataset on post-graduate programmes is more recent and most representative of the current status of health programmes and institutions in the respective countries. The data on health professional programs, I would view as less reliable and more dated. The inclusion of Wikipedia as a validation source is questionable, even university web sites are renowned for being out of date, especially in some African countries were a strong and a centralised higher education management information system is not present. Nevertheless, the authors are transparent in terms of the limitations and share the supplementary material that aids repeatability for those that would want to improve on this baseline. It might be of benefit to start such an exercise by focusing in on one medical programme (MBChB or Nursing for example) and map this throughout the WHO African region in terms of institution and location. The umbrella term of HRH is sufficiently detailed and disaggregated in the paper, however, I am also aware that programme titles often differ and some aspects can overlap in an education programme in one country in comparison to another. It is thus safest to start with such an ambitious mapping exercise, focusing on only a few well defined and standardised programmes. I, however, commend the authors for opening up the debate on the importance of such an exercise by demonstrating the datedness of available data in this regard and highlighting the extreme gaps.

Is the work clearly and accurately presented and does it cite the current literature?

Partly

If applicable, is the statistical analysis and its interpretation appropriate?

Yes

Are all the source data underlying the results available to ensure full reproducibility?

Yes

Is the study design appropriate and is the work technically sound?

Yes

Are the conclusions drawn adequately supported by the results?

Yes

Are sufficient details of methods and analysis provided to allow replication by others?

Yes

Reviewer Expertise:

Human Resources for Health (special focus on medical doctors, nurses and mid-level health providers).

I confirm that I have read this submission and believe that I have an appropriate level of expertise to confirm that it is of an acceptable scientific standard, however I have significant reservations, as outlined above.

# Reviewer report: Reviewer response for version 1

**DOI:** 10.21956/aasopenres.14450.r30478

**Published:** 2024-01-02

**Authors:** Candice Chen

**Affiliations:** 1 Milken Institute School of Public Health, The George Washington University, Washington, District of Columbia, USA

This study represents important work to map and understand health professions education capacity in Africa. The methods section could stand to be expanded along with a limitations section. For example:

 How are post-graduate programs counted? If an institution offers a Master's in Medical Sciences in different specialty areas (e.g., internal medicine, OB/Gyn, Pediatrics, Ophthalmology, etc.) is that counted once or multiple times?

 What does it mean that the "HPP data set was updated and expanded, as practicable"? Was the online search repeated? Over what time period? Or was the update limited to the MDOMS, SANC updates, and the addition of Algerian institutions (and why were Algerian institutions previously excluded)? It is also mentioned in the Discussion that Lusophone PGPs are not included, although this isn't mentioned in the Methods.

 Research productivity is mentioned in the abstract and in the discussion, but how this is measured is not explained in the methods.

 What are the limitations to the online search strategy? In the results section, it was noted that ownership status and founding dates weren't found for some programs - will this also create a challenge for the reported PGPs? It should also be noted that the ANOVA analysis is very limited. Results are unsurprising, but additional information and analysis is needed to fully understand the associations and implications of these associations. An important point made in the Discussion is that this study doesn't capture the capacity (e.g., number of students/graduates) of each institutions/program. This supports the need for future research.

 Overall, this work and the master list of HEIs is an important value added to tracking and understanding health professions education capacity in Africa, particularly the tracking of the multiple professions. As suggested by the authors, it highlights the need for more research and better data. The idea of an open-source on-line database is a worthy goal!

Is the work clearly and accurately presented and does it cite the current literature?

Partly

If applicable, is the statistical analysis and its interpretation appropriate?

Yes

Are all the source data underlying the results available to ensure full reproducibility?

Yes

Is the study design appropriate and is the work technically sound?

Yes

Are the conclusions drawn adequately supported by the results?

Yes

Are sufficient details of methods and analysis provided to allow replication by others?

Partly

Reviewer Expertise:

Health workforce, health services, medical education

I confirm that I have read this submission and believe that I have an appropriate level of expertise to confirm that it is of an acceptable scientific standard, however I have significant reservations, as outlined above.

# Reviewer report: Reviewer response for version 1

**DOI:** 10.21956/aasopenres.14450.r29097

**Published:** 2022-02-09

**Authors:** Jim Campbell, Tapas Nair, Siobhan Fitzpatrick

**Affiliations:** 1 World Health Organization, Geneva, Switzerland

The dataset has immense potential to provide insights into health professionals’ education capacity in Africa, including the proportion of newer schools being privately funded, and the functions of the HEIs in both education and research. This is an important paper in its field.

- p4 - For health workforce stock, WHO’s National Health Workforce Accounts portal has data reported by Member States. https://apps.who.int/nhwaportal/. The majority of AFRO countries have 2020 or 2019 data sources.
- Under methods. Please clarify the period and end point for the update and merging of the HPP and PGP data sets. This will guide the reader on the time point the data reflects. July 2021 is mentioned on page 6.
- p5 – The authors may wish to revisit their position that only ECFMG recognized schools are listed in the WDOMS. The WDOMS lists all schools that are confirmed by the Ministry of Health or Ministry of Education in the country as existing; and then includes *additional* details such as whether the school is operational or not, and the ECFMG recognition status.
- Footnote 24 is unclear – did William Burdick provide the list of schools not included in WDOMS, or did he bring to your attention that there are other schools, which were subsequently researched and identified?
- p5 - It is unclear how the locations of nursing institutions might have changed. Is this because the town name changed, and hence the institution updated its name? How was the differentiation between a school closing in one town and a new one opening in a different town; versus a school relocating?
- The HPP and PGPs included in this dataset is a valuable baseline but should not be considered as comprehensive. Greater clarity on inclusion/exclusion would assist in identifying knowledge gaps. E.g., includes only operational or accredited schools, or all schools for which there is a record of its existence? In the text, nursing schools were excluded if not ‘recognized’ whereas the medical dataset from WDoMS includes both accredited and non-accredited schools, as well as where the information about accreditation is unknown.
- p8, table 2 - the authors may wish to consider health expenditure per capita in PPP to assess the variability between the 47 countries in health spending as a proxy of health education spending.
- p10 - the authors identify challenges in “(a) to establish a managed, open-source of institutions,…., and (b) to develop a visually attractive, user-friendly web-site for trainees,..”. Before creating new mechanisms, could the authors add some consideration of existing data and governance mechanisms in this domain? What exists for countries to capture and report this data through annual administrative processes rather than rely on one-off surveys/research, e.g. the education modules of WHO’s National Health Workforce Accounts? There is scope for instance for this data to be validated and reported by the designated NHWA focal points in the African countries on an annual basis. What are the sectoral challenges to coordinate data across education and health ministries? Are there other regions which have good practice that may be of interest, e.g. annual reporting by the Medical Deans Australia and New Zealand?  

**Copy-editing:**

- p4 - a typo on “SPPS” if referring to the statistical software “SPSS”.
- p5 - two references to MDOMS rather than WDOMS.
- Extra capital letter in the reference to Ijsselmuiden et al. (ref 7).
- First para under the subtitle ‘The WHO African Region’ finishes with two full stops.

Is the work clearly and accurately presented and does it cite the current literature?

Yes

If applicable, is the statistical analysis and its interpretation appropriate?

Partly

Are all the source data underlying the results available to ensure full reproducibility?

Partly

Is the study design appropriate and is the work technically sound?

Yes

Are the conclusions drawn adequately supported by the results?

Partly

Are sufficient details of methods and analysis provided to allow replication by others?

Partly

Reviewer Expertise:

Health workforce

We confirm that we have read this submission and believe that we have an appropriate level of expertise to confirm that it is of an acceptable scientific standard.
